# Dextran and Polymer Polyethylene Glycol (PEG) Coating Reduce Both 5 and 30 nm Iron Oxide Nanoparticle Cytotoxicity in 2D and 3D Cell Culture

**DOI:** 10.3390/ijms13055554

**Published:** 2012-05-09

**Authors:** Miao Yu, Shaohui Huang, Kevin Jun Yu, Alisa Morss Clyne

**Affiliations:** 1 Mechanical Engineering and Mechanics Department, Drexel University, Philadelphia, PA 19104, USA; E-Mail: my87@drexel.edu (M.Y.); 2 Institute for Environmental Medicine and Department of Physiology, School of Medicine, University of Pennsylvania, Philadelphia, PA 19104, USA; E-Mails: shaohuih@mail.med.upenn.edu (S.H.); kevinyu2@mail.med.upenn.edu (K.J.Y.)

**Keywords:** endothelial cells, cytotoxicity, superparamagnetic iron oxide nanoparticles, dextran, PEG, reactive oxygen species, 3D cell culture

## Abstract

Superparamagnetic iron oxide nanoparticles are widely used in biomedical applications, yet questions remain regarding the effect of nanoparticle size and coating on nanoparticle cytotoxicity. In this study, porcine aortic endothelial cells were exposed to 5 and 30 nm diameter iron oxide nanoparticles coated with either the polysaccharide, dextran, or the polymer polyethylene glycol (PEG). Nanoparticle uptake, cytotoxicity, reactive oxygen species (ROS) formation, and cell morphology changes were measured. Endothelial cells took up nanoparticles of all sizes and coatings in a dose dependent manner, and intracellular nanoparticles remained clustered in cytoplasmic vacuoles. Bare nanoparticles in both sizes induced a more than 6 fold increase in cell death at the highest concentration (0.5 mg/mL) and led to significant cell elongation, whereas cell viability and morphology remained constant with coated nanoparticles. While bare 30 nm nanoparticles induced significant ROS formation, neither 5 nm nanoparticles (bare or coated) nor 30 nm coated nanoparticles changed ROS levels. Furthermore, nanoparticles were more toxic at lower concentrations when cells were cultured within 3D gels. These results indicate that both dextran and PEG coatings reduce nanoparticle cytotoxicity, however different mechanisms may be important for different size nanoparticles.

## 1. Introduction

Superparamagnetic iron oxide nanoparticles are widely used in biomedical applications such as drug delivery [1], magnetic resonance imaging [2], magnetic hyperthermia [3], and cell labeling and separation [4]. The nanoparticles can be manipulated using an external magnetic field, yet they do not retain their magnetic properties when the magnetic field is removed [5]. Our long-term goal is to use iron oxide nanoparticles as drug carriers for atherosclerosis treatment; however, before we develop this drug delivery platform, we must determine the endothelial toxicity of the nanoparticles themselves. The endothelium could also be an important drug delivery target for promoting or inhibiting angiogenesis in wound healing or cancer, respectively [6]. In addition, when these nanoparticles are injected into the bloodstream for imaging studies or drug delivery to other organs, they will first make contact with the endothelium before subsequently reaching the targeted tissue. Iron oxide nanoparticle interactions with endothelial cells are therefore of particular importance and interest. Bare iron oxide nanoparticles are cytotoxic. Cell viability and metabolic activity decrease significantly when cells are exposed to high iron oxide nanoparticle concentrations [7–10]. The best developed theory to explain nanoparticle-induced cytotoxicity is reactive oxygen species (ROS) generation. ROS are normally formed *in vivo* at low levels for cell signaling or at higher levels by macrophages and neutrophils fighting infection [11]. In these conditions, ROS are quickly neutralized by antioxidant defenses [12]. ROS are thought to be induced by iron oxide nanoparticles through a combination of NADPH oxidase during endocytotosis, direct formation of free radicals on the nanoparticle surface, and catalysis to more reactive ROS forms via the Fenton reaction [13]. As nanoparticle-induced ROS rise with increasing nanoparticle concentration, these ROS can cause damage to the cell membrane, DNA, and ROS-mediated signal transduction [14]. Nanoparticle-induced ROS have also been shown to alter the actin cytoskeleton and cell stiffness [15]. This effect may feed back on itself, since decreased actin dynamics induce mitochondrial membrane depolarization and further increase the ROS production resulting in cell death [16].

Iron oxide nanoparticles are generally coated to reduce aggregation and cytotoxicity [17]. Dextran (C_6_H_10_O_5_), a branched polysaccharide, is commonly used to coat nanoparticles. In solution, dextran interacts with the metal nanoparticle surface to form 20 to 150 nm coated aggregates [18]. Dextran coated iron oxide nanoparticles have been used for many purposes, including as MRI contrast agents, to investigate nanoparticle accumulation and cellular uptake in malignant neoplasms *in vivo*, and to transform nanoparticles into active, targeted probes [19–21]. Polyethylene glycol (PEG) is a hydrophilic polymer that is stable, biocompatible, and used in many drug and gene delivery applications [22]. PEG coatings have been used to reduce phagocytic capture of nanoparticles by the immune system, which can extend nanoparticle circulation time and subsequent accumulation in targeted tissue [23].

Nanoparticle size plays an important role in nanoparticle cellular uptake. *In vivo* experiments showed that as the diameter of superparamagnetic magnetite-dextran nanoparticles increased, the liver uptake also increased [24]. Similarly, larger nanoparticles improved cell uptake of carboxydextran-coated iron oxide nanoparticles, which enhanced cell tagging and lipofection-based methods [25]. Nanoparticle size, in addition to structure and surface coating, affects cytotoxicity. However, so far there are inconsistent conclusions as to whether large or small nanoparticles induce higher nanoparticle cytotoxicity. For nickel ferrite nanoparticles tested in neuroblastoma cells, larger nanoparticles (150 ± 50 nm diameter) induced higher cytotoxicity than smaller particles (10 ± 3 nm diameter) [26]. Similarly, silver nanoparticles (<100 nm) were less toxic to Drosophila eggs than those greater than 100 nm in size [27]. In other studies, smaller silver nanoparticles (10 nm) induced a greater apoptotic effect in osteoblasts than larger nanoparticles (50 and 100 nm), and 21 nm silica nanoparticles were less toxic than 48 nm nanoparticles in myocardial cells [28]. Therefore the relationship between nanoparticle size and cell toxicity remains an important area of study.

While iron oxide nanoparticles and their cytotoxic effects are widely studied *in vitro* and *in vivo*, much remains to be understood regarding the effect of both nanoparticle size and coating on toxicity mechanisms.In this study, we coated 5 and 30 nm nanoparticles with dextran and PEG and investigated cytotoxicity, ROS formation, and actin cytoskeleton disruption in endothelial cells. We further compared cytotoxicity in 2D cell culture to cells suspended in a 3D hydrogel. Our data show that while iron oxide nanoparticle coatings decrease cytotoxicity, the mechanism may vary for 5 and 30 nm nanoparticles. In addition, nanoparticles are toxic at lower concentrations in 3D culture. This study clarifies how nanoparticle size and coating affect cytotoxicity in endothelial cells and will contribute to enhanced nanoparticle design for biomedical applications.

## 2. Results and Discussion

### 2.1. Nanoparticle Coating

**Figure 1:**
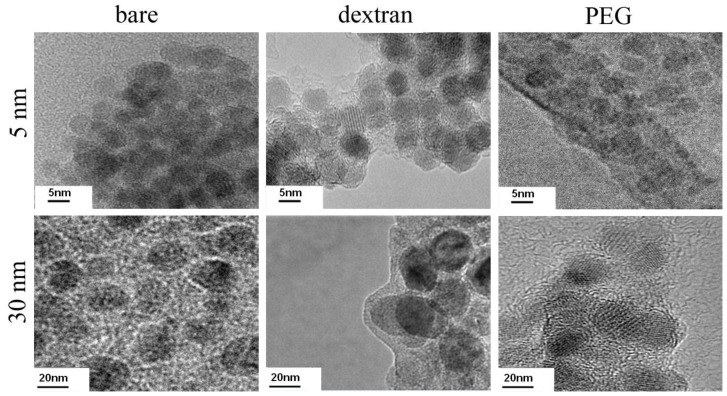
5 and 30 nm nanoparticles were coated with dextran and polymer polyethylene glycol (PEG). Nanoparticle samples were sonicated for 1 h with either dextran or m-PEG-silane and imaged by TEM.

The objective of this study was to determine how different nanoparticle coatings affect cytotoxicity mechanisms for two different nanoparticle sizes. We first verified that nanoparticles could be coated with both dextran and PEG. After 1 h of coating, a thin dextran or PEG layer formed on the nanoparticle surface for both 5 nm and 30 nm nanoparticles (Figure 1). For 5 nm particles, the average layer thickness was around 2 nm, whereas for 30 nm particles, the coating layer thickness was slightly larger at around 5 nm. Coating layer thickness increased with longer coating times. Nanoparticle coating stability was confirmed for up to three months of storage at 4 °C by TEM. All nanoparticles in these experiments were used within one month of coating, and no changes in coated nanoparticle cellular effects were observed over this storage time.

### 2.2. Cell Uptake of Iron Oxide Nanoparticles

**Figure 2:**
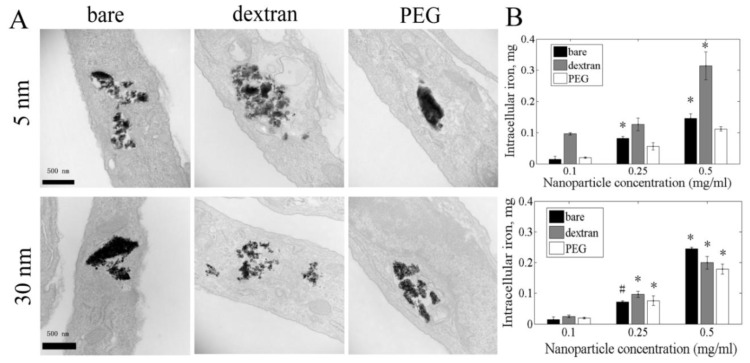
Iron oxide nanoparticles were taken into cells after 3 h incubation. (**A**) Porcine aortic endothelial cells (PAEC) were incubated with 0.1 mg/mL bare, dextran or PEG coated iron oxide nanoparticles. Large nanoparticle aggregation was observed in vesicles in the cytoplasm; (**B**) Intracellular iron increased with nanoparticle concentration, as measured by iron absorbance. ^*^ *p* < 0.01 compared to 0.1 mg/mL for same coating condition, # *p* < 0.05 compared to 0.1 mg/mL for same coating condition.

Cell nanoparticle uptake was measured qualitatively by TEM and quantitatively by iron absorbance. After 3 h, bare and coated nanoparticles were taken into cells and clustered in cytoplasmic vacuoles (Figure 2). No nanoparticles were observed in cell nuclei. Intracellular iron concentration increased in a nanoparticle dose-dependent manner for both 5 and 30 nm nanoparticles. For 5 nm nanoparticles, dextran coated nanoparticles showed the highest cellular uptake for each concentration. At 0.5 mg/mL, cells incubated with dextran coated nanoparticles had more than 50% more intracellular iron than cells incubated with bare and PEG coated nanoparticles. However, for 30 nm nanoparticles, cells took up more bare nanoparticles than coated ones. Cells incubated with 0.5 mg/mL bare nanoparticles had 20.2% and 26.6% more intracellular iron than cells incubated with dextran or PEG coated nanoparticles.

### 2.3. Cytotoxicity

**Figure 3:**
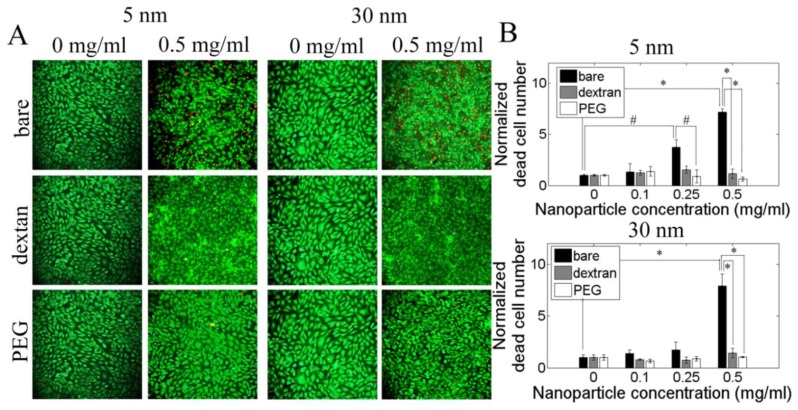
Dextran and PEG coating reduced iron oxide nanoparticle cytotoxicity, as measured by a Live/Dead assay. PAEC were incubated with 0, 0.1, 0.25 and 0.5 mg/mL of 5 and 30 nm bare and coated nanoparticles for 24 h. (**A**) Selected fluorescent images in which green = live cells and red = dead cells; and (**B**) Quantification of dead cell number by Image J. ^*^ *p* < 0.01, # *p* < 0.05.

Both 5 nm and 30 nm bare nanoparticles decreased cell viability as measured by a Live/Dead assay. Significant cell death occurred when cells were exposed to 0.5 mg/mL bare nanoparticles of either size, with a more than six fold increase in dead cells compared to control cells that were not exposed to nanoparticles (Figure 3). However, cell viability remained the same at all nanoparticle concentrations for nanoparticles coated with dextran and PEG. Thus nanoparticle coating decreased cytotoxicity, but nanoparticle size did not have an effect.

### 2.4. ROS Formation

**Figure 4:**
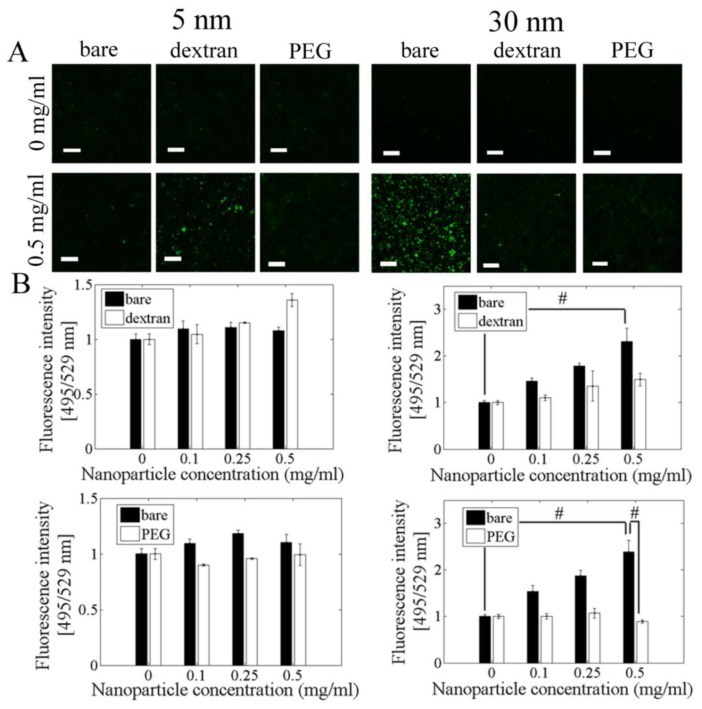
Bare 30 nm nanoparticles induced the highest level of intracellular ROS formation. (**A**) Selected confocal microscopy ROS images. PAEC were incubated with different concentrations of bare and coated nanoparticles for 3 h. Cells were then labeled with 10 μM carboxy-H_2_DCFDA (green), a general ROS indicator. Scale bar = 100 μm; (**B**) Quantification of ROS formation by Image J. # *p* < 0.05.

Cells loaded with 30 nm bare nanoparticles showed the highest ROS formation after 3 h of nanoparticle exposure for all concentrations. At all nanoparticle concentrations, there was minimal ROS formation with 5 nm nanoparticles. However, dextran coated 5 nm nanoparticles showed the most ROS formation at 0.5 mg/mL, with 23.1% more than cells with no nanoparticles. For 30 nm bare nanoparticles, ROS fluorescence intensity increased by 56.5% for 0.5 mg/mL nanoparticles as compared to cells without any nanoparticles. Dextran coating decreased ROS fluorescent intensity by 35.2% and PEG coating decreased ROS fluorescent intensity by 62.6% at 0.5 mg/mL nanoparticle concentration (Figure 4).

### 2.5. Cell Length and Actin Cytoskeleton

**Figure 5:**
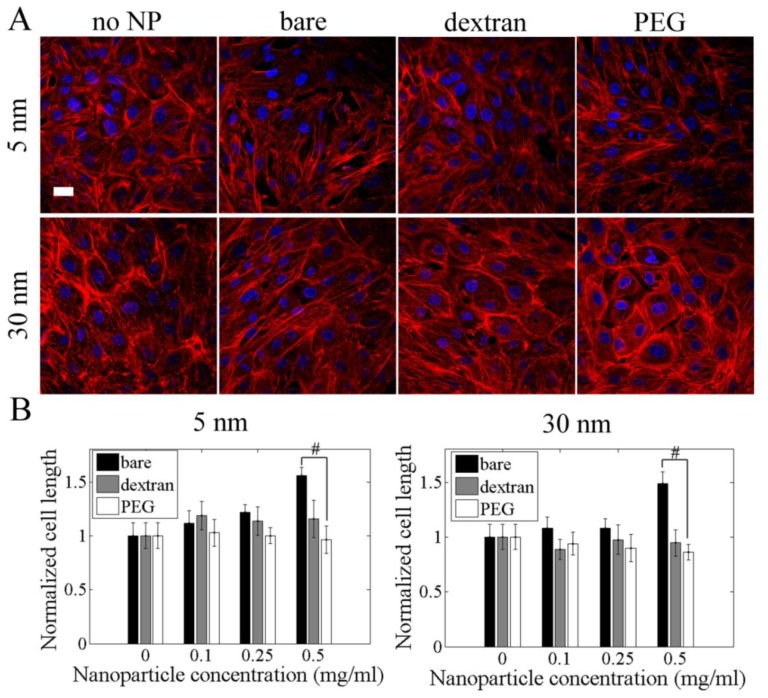
Dextran and PEG coated nanoparticles reduced cell elongation and stress fiber formation. (**A**) Selected confocal images of cells labeled for actin. PAEC were incubated with 0.5 mg/mL bare and coated 5 and 30 nm nanoparticles for 24 h. Samples were then fixed with paraformaldehyde, permeabilized with Triton X-100, and labeled with rhodamine phalloidin (actin, red) and Hoechst (nuclei, blue). Scale bar = 20 μm; (**B**) Cell length was quantified by Image J. # *p* < 0.05.

Cell length increased by 40–60% when cells were exposed to 0.5 mg/mL bare 5 or 30 nm nanoparticles. Actin cytoskeleton disruption was observed in PAEC loaded with 0.1, 0.25 and 0.5 mg/mL bare nanoparticles for both 5 nm and 30 nm after 24 h. Cells with bare nanoparticles were more elongated compared to cells with no nanoparticles (Figure 5). Dextran and PEG coated nanoparticles had no effect on cell length and did not show actin cytoskeleton disruption.

### 2.6. 3D Cell Culture

**Figure 6:**
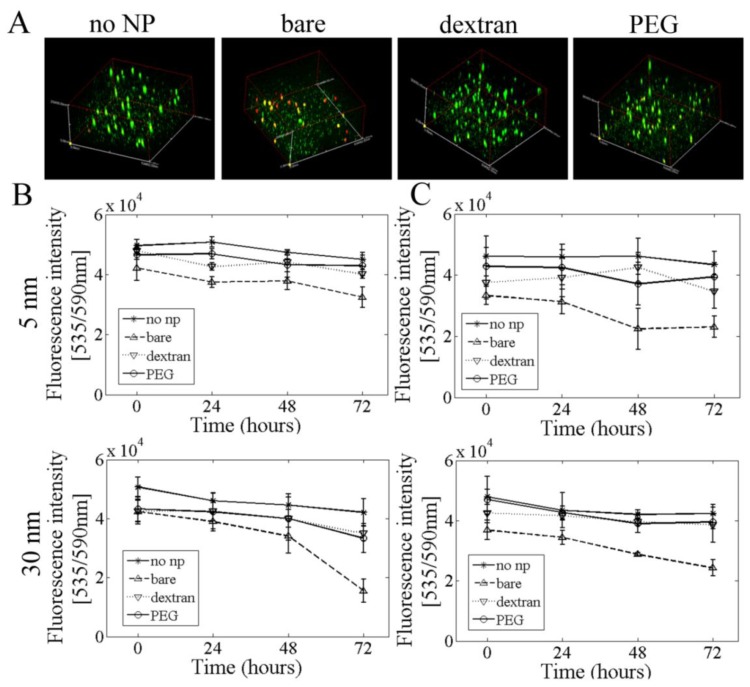
Dextran and PEG nanoparticle coating improved cell viability in 3D culture. (**A**) Selected confocal images of PAEC viability in 3D alginate constructs with nanoparticles in the alginate, as measured by the Live/Dead assay. Alginate was mixed with 0.1 mg/mL bare and coated nanoparticles and cells. Alginate-nanoparticle-cell constructs were labeled using a Live/Dead assay. Cell viability in 3D constructs with (**B**) nanoparticles in the alginate and (**C**) nanoparticles inside cells was measured using an Alamar blue assay.

Nanoparticle toxicity occurred at a lower nanoparticle concentration in 3D *vs*. 2D cell culture. When cells were cultured in alginate scaffolds with either 0.1 mg/mL nanoparticles in the alginate or nanoparticles pre-loaded inside cells, bare nanoparticles had the highest degree of cell toxicity for both 5 and 30 nm nanoparticles. Similarly, cell toxicity increased with time for cells exposed to bare nanoparticles. However, dextran and PEG coated nanoparticles did not show significantly reduced cell viability at four time points (Figure 6).

### 2.7. Discussion

Iron oxide nanoparticles may be useful across a wide variety of medical applications, including magnetic resonance imaging contrast enhancement, immunoassays, and drug delivery [29]. However, many aspects of nanoparticle-induced cell toxicity remain unclear. In this study, we showed that both 5 and 30 nm bare iron oxide nanoparticles decreased endothelial cell viability. Interestingly, only 30 nm bare nanoparticles caused a dose dependent increase in ROS formation, whereas both sizes induced cell elongation with actin stress fiber formation and eventually cell death. When endothelial cells were exposed to nanoparticles of either size coated with dextran or PEG, cell viability was maintained especially at higher nanoparticle concentrations. Nanoparticle coating effects were similar for cells in both 2D and 3D cell culture systems, although lower nanoparticle concentrations were cytotoxic in 3D culture. These data suggest that ROS formation contributes to iron oxide nanoparticle toxicity for larger particles and can be significantly reduced using biocompatible nanoparticle coatings; however, alternative toxicity mechanisms may similarly be reduced by nanoparticle coatings for smaller particles.

Nanoparticle polysaccharide and polymer coatings can reduce nanoparticle aggregation as well as enhance biocompatibility [30]. Dextran and PEG coated nanoparticles tend to form homogenous suspensions due to anisotropic dipolar attraction and high surface-to-volume ratios [31]. We still observed nanoparticle aggregation in our TEM samples for nanoparticles coated with dextran and PEG, although nanoparticles coated with dextran appeared more dispersed in solution. It remains unclear if aggregation consistently occurred in solution, during the TEM drying process, or after cell uptake. When we repeated our cytotoxicity experiments with nanoparticles that were coated with dextran during synthesis, which should be more dispersed than our nanoparticles which were coated after synthesis, we observed similar toxicity results. However, nanoparticle aggregation can be decreased by controlling coating properties, for example by raising the weight ratio of dextran to iron oxide nanoparticles [31].

TEM images of cells exposed to 5 and 30 nm nanoparticles show nanoparticles clustered in large vacuoles, which may have formed by merging smaller vacuoles. No nanoparticles were found in cell nuclei, even though the 5 nm particles are smaller than the nuclear pore opening (~ 9 nm). This may be due to nanoparticle clustering or rapid nanoparticle isolation in vacuoles. Nanoparticle cellular uptake can be affected by many factors, including size, shape, surface charge and functional groups [32]. In our experiments, intracellular nanoparticles increased with nanoparticle medium concentration for all sizes and coatings. However, with the exception of 5 nm dextran coated nanoparticles, the 30 nm nanoparticles showed higher internalization at 3 h than the 5 nm nanoparticles. Previous studies similarly demonstrated that under the same medium concentration, larger nanoparticles led to higher cell internalization when compared with smaller nanoparticles. These larger particles, however, had a slower uptake rate [33]. Interestingly, 5 nm dextran coated nanoparticles had the largest cell uptake at each concentration. This may be because dextran decreased nanoparticle aggregation, which led to faster uptake. However, no significant changes were observed for cell nanoparticle uptake at later time points, suggesting that the majority of uptake occurred within the first three hours. Additional studies are needed to confirm if 5 nm dextran coated nanoparticles were taken up via a different mechanism than the other nanoparticles.

The most likely mechanism for iron oxide nanoparticle cytotoxicity in the literature is ROS formation [15,34]. *In vivo*, most ROS are formed as by-products of mitochondrial electron transport or via NADPH oxidase, xanthine oxidase, and nitric oxide synthase [35–37]. Superoxide (O_2_^−^) is formed by one electron reduction of O_2_, and further reduction of oxygen (catalyzed by superoxide dismutase) leads to hydrogen peroxide (H_2_O_2_) formation. Hydrogen peroxide can either be converted to inert water and oxygen by catalase, or it can be converted to the highly reactive and damaging hydroxyl radical (OH·) in the presence of metal ions through the Fenton reaction [35]. Throughout the cell, antioxidant enzymes such as glutathione (GSH) maintain the cell in a state of low oxidative stress. For iron oxide nanoparticle-induced ROS, the initial reactive species are likely formed through NADPH-oxidase activation or stabilization during nanoparticle endocytosis. Superoxide and hydrogen peroxide are then converted to the more damaging hydroxyl radicals through reactions with the iron. The large ROS load may overwhelm the cell’s protective antioxidants [38], and nanoparticle-induced ROS will attack lipids, polysaccharides, proteins and DNA, causing cell injury and cell death [15].

Our data show that for 30 nm nanoparticles, both dextran and PEG coating reduce ROS formation and cell toxicity. Dextran and PEG coating block ROS interaction with iron oxide nanoparticles, which may prevent the Fenton reaction from occurring and allow the cell’s antioxidant defense to neutralize ROS before they become dangerous hydroxyl radicals. Our data further show that 5 nm bare or coated nanoparticles do not induce significant ROS formation. This is contrary to our expectations that since nanoparticles with a smaller size have a larger surface area/volume ratio, there would be more surface interaction of H_2_O_2_ with iron, leading to more OH·formation [34]. These small nanoparticles may not induce the initial ROS formation via NADPH oxidase during endocytosis, or they may produce reactive OH· so quickly that by 3 h the ROS levels are already decreasing. In our previous work, we showed that ROS levels increased up to 3 h and remained constant, but those experiments were performed with 20–40 nm nanoparticles. Additional investigation is needed to determine the relationship between 5 nm nanoparticles and ROS. If these small nanoparticles do not cause ROS production, then the bare nanoparticles likely induce a different cell toxicity mechanism that is prevented by both dextran and PEG coating.

The cytoskeleton is a dynamic network consisting of actin polymers, microtubules, and associated proteins [39]. Actin in particular plays an important role in cell shape, adhesion, and motility [40,41]. Increasing evidence shows that the actin cytoskeleton is essential to endocytotic processes, including pseudopod extension, phagocytotic engulfment, and cell surface remodeling for vesicle formation and movement [42]. In our experiments, cells exposed to higher concentrations (0.5 mg/mL) of bare nanoparticles showed significant cell elongation and actin cytoskeleton disruption. While these elongated cells have not yet initiated a cell death pathway, since dying cells would retract and round up, they exhibit a significantly stressed morphology. This could have been a result of iron oxide nanoparticle-induced ROS formation, which alters the cytoskeleton and increases cell permeability and microtubule remodeling [43]. These processes can redirect actin cytoskeleton polymerization and contraction [44]. These cells may recover from the nanoparticle-induced oxidative stress, or they may later progress down a cell death pathway.

The majority of nanotoxicity studies have been performed in 2D cell culture. We investigated whether nanoparticles would show altered toxicity when cells were suspended in a 3D gel. A sodium alginate polymer was used because it is nontoxic, biocompatible, and well characterized by our lab and others. Alginate forms a gel in the presence of calcium, and since cells do not specifically attach to alginate, they do not proliferate in culture which allows isolation of toxicity effects [45]. Similar to 2D culture results, cells incubated with bare nanoparticles had the lowest viability after 72 h, while dextran and PEG coated nanoparticles maintained cell viability over time. However, nanoparticles were more toxic at lower concentration in 3D culture. In 2D culture, bare nanoparticles showed significant cytotoxicity at 0.5 mg/mL, while in 3D culture bare nanoparticles were cytotoxic at 0.1 mg/mL. This may be due to increased contact area between nanoparticles and cells in 3D culture [46].

Our experiments showed that polysaccharide and polymer coatings reduce nanoparticle cytotoxicity independent of nanoparticle size; however our research is not without limitations. While we extensively sonicated our nanoparticles at each experimental stage, and attempted to select monodispersed samples, bare nanoparticle aggregation made it difficult to evenly coat individual nanoparticles and may obscure subtle size-specific nanoparticle effects. However we did observe single nanoparticles in TEM images of nanoparticles alone and nanoparticles inside cells. We further observed similar cytotoxicity results when experiments were repeated with nanoparticles that were synthesized and coated simultaneously (and therefore more dispersed).Our studies were performed *in vitro*, independent of many *in vivo* conditions including plasma proteins and shear stress from blood flow. Future work will include more detailed *in vitro* experimentation as well as animal studies to understand potentially different *in vivo* toxicity mechanisms. While we believe that nanoparticles are taken up by cells through endocytosis, we do not know the effect of different endocytotic mechanisms on ROS formation or cell toxicity. Moreover, we used general ROS indicators and inhibitors, and therefore did not determine the type of ROS responsible. More specific indicators and inhibiters will be used in the future.

Many papers have recently been published regarding iron oxide nanoparticle cytotoxicity in different cell systems and with different nanoparticle sizes and coatings. For example, both dextran and lipid coatings have been shown to decrease iron oxide nanoparticle cytotoxicity in endothelial cells, and very low iron oxide nanoparticle concentrations (that do not induce oxidative stress and toxic effects) may negatively impact DNA stability [47–50]. Since each paper differs in method and scope, direct comparisons and generalizations are difficult. Yet each study contributes to our understanding of cellular nanotoxicity mechanisms and expands our repertoire of nanoparticle modifications that limit cytotoxic effects. Our research in particular highlights that both dextran and PEG coatings can decrease ROS-induced nanoparticle toxicity, toxicity mechanisms may differ depending on nanoparticle size, and cytotoxicity may increase for cells in 3D culture.

## 3. Experimental Section

### 3.1. Cell Culture

Porcine aortic endothelial cells (PAEC) were isolated from porcine aortae and cultured in low glucose Dulbecco’s modified Eagle’s medium (DMEM) supplemented with 5% fetal bovine serum and 1% penicillin-streptomycin. Cells (passage 5 to 8) were maintained in a 37 °C, 5% CO_2_ incubator, and medium was changed every two days. For 2D cell culture, PAEC were seeded in a 24-well plate at 200,000 cells/well and cultured for two days. One milliliter nanoparticle solution for each coating and concentration was then added to cells for 3–24 h. For 3D cell culture, sodium alginate (1% *w/v*, FMC BioPolymer, Philadelphia, PA, USA) was used as the scaffold material. 0.1 mg/mL 5 or 30 nm bare, dextran, or PEG coated nanoparticles were either mixed into the alginate solution at the same time as cells (1.5 × 10^5^ cells/mL), or nanoparticles were incubated with cells for 24 h after which the trypsinized cells with internalized nanoparticles were mixed in the alginate solution. 0.3 g alginate was then deposited into a 6-well plate and incubated with 5% calcium chloride (CaCl_2_) as the ionic cross-linking solution for 5 min, after which supplemented medium was added and samples were stored in the incubator. Medium was changed every 2 days to maintain cell viability.

### 3.2. Nanoparticle Coating

Five and thirty nanometer diameter bare iron oxide nanoparticles were purchased from NN-Labs (Fayetteville, AR, USA). For dextran coated nanoparticles, 10 mg dextran (MW 6000, Sigma, St. Louis, MO, USA) was added to 10 mg iron oxide nanoparticles in 20 mL 0.5 M NaOH and sonicated with a Sonicator 3000 (Qsonica, Newton, CT, USA) for 0.5 to 1.5 h. Coated nanoparticles were then dialyzed using a 12,000–14,000 MW Spectra/Por Dialysis membrane (Spectrum Laboratories, Rancho Dominguez, CA, USA) for 24 h in 1.5 L distilled water to remove excess dextran [51]. For PEG coating, iron oxide nanoparticles were washed with ethanol and dried in an Isotemp oven (Fisher Scientific, Houston, TX) at 100 °C for 30 min. Ten milligram iron oxide nanoparticles were mixed with 5 mL of 3 mM methoxy-PEG-silane (MW 5000, Laysan BioInc, Arab, AL, USA) and sonicated for 1 h [52]. The mixture was washed thoroughly with ethanol, and centrifuged for 5 min at 5000 rpm. The supernatant was then removed and nanoparticles were resuspended in cell culture medium.

### 3.3. Nanoparticle Coating and Cell Uptake by Transmission Electron Microscopy (TEM)

Dextran and PEG coatings were verified by TEM. Nanoparticle solutions were diluted in distilled water, and a single drop of nanoparticle solution was added to a copper TEM grid (Pacific Grid-Tech, San Francisco, CA, USA). After each sample was dried at room temperature for 24 h, nanoparticles were imaged with a JEOL JEM100CX TEM at 100 kV. To determine cell nanoparticle uptake, increasing nanoparticle concentrations were added to confluent PAEC (2 × 10^5^ cells/mL) in a 24 well plate for 3 h. Samples were washed with phosphate buffered saline (PBS), fixed with 4% paraformaldehyde and 2.5% glutaraldehyde for 15 min, and cells were then gently scraped from the dish and kept in fixative for another 2 h on ice. Cells were then pelleted and washed with 0.1 M sodium cacodylate buffer. The cell pellets were mixed with warmed 3% agarose liquid, and subsequently the cooled agarose gel pellets were sliced into 1 mm thick bricks and post fixed with 2% osmium tetroxide and 0.5% uranyl acetate. Agarose bricks were dehydrated with graded acetone, embedded in Epon, polymerized in a 60 °C oven for 2 days, and cut into ultrathin (100 nm) sections for TEM.

Cell nanoparticle uptake was quantified by dissolving cells and their intracellular nanoparticles and measuring iron absorbance. Cells incubated with iron oxide nanoparticles were trypsinized, and 200 μL cell solution was incubated with 200 μL of 1 M NaOH for 30 min at 90 °C. Five hundred microliter of 1 M HCl was then added for 2–3 h to completely dissolve the nanoparticles. Two hundred microliter cell lysate was transferred to 96-well-plate, and absorbance (335 nm) was measured with GENios microplate reader.

### 3.4. Cell Viability

Cell viability was assessed via a Live/Dead assay (Invitrogen, Grand Island, NY, USA). In the presence of intracellular esterases in live cells, nonfluorescent calcein AM is converted to fluorescent calcein (green). In dead cells, ethidium homodimer-1 (EthD-1) enters through damaged cell membranes and fluoresces when it binds to nucleic acids (red). Confluent PAEC in 24 well plates were incubated with increasing concentrations of bare and coated 5 and 30 nm nanoparticles for 24 h. Two micromole per liter calcein AM and 4 μM EthD-1 were added to each well for 30 min, after which cells were imaged in an Olympus IX81 inverted fluorescent microscope. For 3D samples, alginate-nanoparticle-cell constructs were incubated with 500 μL of Live/Dead solution for 30 min and then placed on a coverslip just prior to imaging with an Olympus IX81 confocal microscope. A depth of 250 μm was scanned in the gel for 3D Live/Dead images.

An Alamar blue assay, which measures cell mitochondrial metabolic activity, was used to confirm cell viability in 3D samples [53]. In this assay, the tetrazolium-based dye resazurin is non-fluorescent blue until it is reduced in mitochondria to fluorescent red. Alginate-nanoparticle-cell samples were incubated with 2 mL medium with 200 μL Alamar blue (AbD Serotec, Raleigh, NC, USA). After 4 h, 400 μL medium from each sample was measured for fluorescence intensity using a GENios microplate reader (excitation/emission: 535/590 nm) [46].

### 3.5. Reactive Oxygen Species (ROS)

ROS in live cells were detected with the Image-iT Green Reactive Oxygen Species kit (Invitrogen), which uses 5-(and-6)-carboxy-2′,7′-dichlorodihydrofluorescein diacetate (carboxy-H_2_DCFDA) as a general ROS fluorogenic indicator. Tert-butyl hydroperoxide (tBHP) was the positive control, and Hoechst 33342 was used to label cell nuclei. Confluent PAEC in 24 well glass bottom dishes were incubated with different concentrations of bare and coated 5 and 30 nm nanoparticles for 3 h. Cells were labeled with 10 μM carboxy-H_2_DCFDA and 1.0 mM Hoescht according to the manufacturer’s protocol. Samples were then imaged in an Olympus IX81 confocal microscope (excitation/emission 495/529 nm and 350/461 nm for carboxy-H_2_DCFDA and Hoechst respectively).

### 3.6. Actin Cytoskeleton

The actin cytoskeleton was labeled to assess changes in cell shape with nanoparticle exposure. PAEC were cultured in 24 well plates for 24 h, after which bare and coated 5 and 30 nm iron oxide nanoparticles were added for another 24 h. Samples were then fixed with 4% paraformaldehyde, permeabilized with 1% *v/v* Triton X-100, and incubated with rhodamine phalloidin (1 unit/well, actin) in 1% bovine serum albumin (BSA, Sigma) followed by Hoechst (1 μg/mL, nuclei). Samples were imaged in by confocal microscopy (excitation/emission: 540/565 for rhodamine phallodin). Cell length was measured with Image-J by selecting the distance between the two opposing end points of one cell.

### 3.7. Statistical Analysis

Data are graphed as mean ± standard deviation. Statistical analyses were performed with Matlab and Excel. Comparisons between two groups were analyzed using Student’s *t*-test with statistical significance at *p* < 0.05 (#) or *p* < 0.01 (^*^). Experiments were performed in triplicate, and each experiment was repeated at least 3 times.

## 4. Conclusions

While iron oxide nanoparticles for *in vivo* applications are coated for safety, these coatings will likely degrade in the body or in the environment. It is critical to understand how bare nanoparticles interact with cells to determine the effect of those nanoparticles that lose their coating either extracellularly or intracellularly [8]. We now show that both dextran and PEG coating decrease nanoparticle cytotoxicity, but that cytotoxicity mechanisms may vary for different sized nanoparticles. In addition, since nanoparticles were more toxic in 3D culture, these types of *in vitro* systems should be considered in future cytotoxicity studies.
